# Improved Ca^2+^ release synchrony following selective modification of I_tof_ and phase 1 repolarization in normal and failing ventricular myocytes

**DOI:** 10.1016/j.yjmcc.2022.07.009

**Published:** 2022-11

**Authors:** Ewan D. Fowler, Nan Wang, Melanie J. Hezzell, Guillaume Chanoit, Jules C. Hancox, Mark B. Cannell

**Affiliations:** a School of Physiology, Pharmacology & Neuroscience, Faculty of Biomedical Sciences, University of Bristol, University Walk, Bristol BS8 1TD, UK; b University of Bristol Veterinary School, Langford, Bristol BS40 5DU, UK

**Keywords:** Electrophysiology, Heart failure, Arrhythmias, Sudden cardiac death, Calcium cycling/excitation-contraction coupling

## Abstract

Loss of ventricular action potential (AP) early phase 1 repolarization may contribute to the impaired Ca^2+^ release and increased risk of sudden cardiac death in heart failure. Therefore, restoring AP phase 1 by augmenting the fast transient outward K^+^ current (I_tof_) might be beneficial, but direct experimental evidence to support this proposition in failing cardiomyocytes is limited. Dynamic clamp was used to selectively modulate the contribution of I_tof_ to the AP and Ca^2+^ transient in both normal (guinea pig and rabbit) and in failing rabbit cardiac myocytes. Opposing native I_tof_ in non-failing rabbit myocytes increased Ca^2+^ release heterogeneity, late Ca^2+^ sparks (LCS) frequency and AP duration. (APD). In contrast, increasing I_tof_ in failing myocytes and guinea pig myocytes (the latter normally lacking I_tof_) increased Ca^2+^ transient amplitude, Ca^2+^ release synchrony, and shortened APD. Computer simulations also showed faster Ca^2+^ transient decay (mainly due to fewer LCS), decreased inward Na^+^/Ca^2+^ exchange current and APD. When the I_tof_ conductance was increased to ~0.2 nS/pF in failing cells (a value slightly greater than seen in typical human epicardial myocytes), Ca^2+^ release synchrony improved and AP duration decreased slightly. Further increases in I_tof_ can cause Ca^2+^ release to decrease as the peak of the bell-shaped I_Ca_-voltage relationship is passed and premature AP repolarization develops. These results suggest that there is an optimal range for I_tof_ enhancement that may support Ca^2+^ release synchrony and improve electrical stability in heart failure with the caveat that uncontrolled I_tof_ enhancement should be avoided.

## Highlights

• Loss of fast transient outward current alters phase 1 of the cardiac AP in HF.
• By computing and injecting a synthetic transient outward current phase 1 was altered.
• Moderate increases in transient outward current improves Ca release synchrony.
• Reduced arrhythmogenic late Ca sparks in heart failure accompany phase 1 restoration
• There is a limited therapeutic window for transient outward current augmentation.

## Graphical abstract

**Figure:**
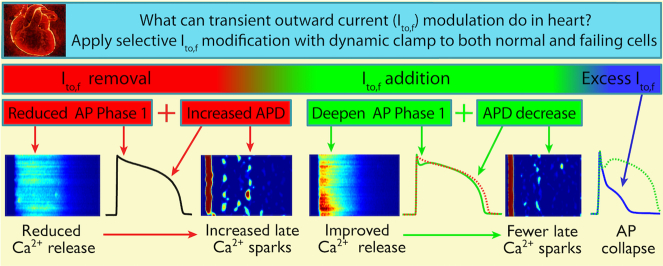
Graphical abstract

## 1 Introduction

The human ventricular action potential (AP) exhibits a characteristic spike-and-dome morphology due to rapid phase 1 repolarization. Phase 1 is dominated by a fast activating and inactivating transient outward K^+^ current (I_tof_) which is decreased in HF [[1], [2], [3]]. This reduction in I_tof_ is related to the (as yet unexplained) loss of pore-forming Kv4.3 alpha-subunits and ancillary proteins [3]. The reduction in the depth [4] and rate [5] of phase 1 repolarization impacts peak inward Ca^2+^ current (I_Ca_) to produce a more heterogeneous Ca^2+^ release [6] and a smaller Ca^2+^ transient in failing myocytes [7]. This loss of Ca^2+^ release synchrony, coupled with other deleterious effects such as t-tubule changes [8,9] and ventricular remodelling [10], may help explain the decreased contractile performance seen in heart failure [11].

Augmenting early repolarization by enhancing I_tof_ has been proposed as a possible therapy in heart failure to improve contractility [4,12]. However, when I_tof_ was augmented electrophysiologically with dynamic clamp (DyC) the Ca^2+^ transient amplitude and cell shortening decreased in non-failing canine endocardial myocytes [13]. These observations led to the suggestion that I_tof_ could be a “negative regulator of contractility” [13]. Similarly, computer simulations predicted that restoring the phase 1 notch in failing canine epicardial cells might only slightly improve Ca^2+^ transient amplitude [14]. In contrast to these results, we recently showed that introducing a normal AP with a phase 1 notch (via voltage clamp) appeared to increase Ca^2+^ transient amplitude in rabbit myocytes [15].

The ability of an augmented I_tof_ to increase AP phase 1 notch depth in failing human ventricular myocytes has been confirmed by DyC, although effects on Ca^2+^ release and contractility were not measured [16]. In this study we combined confocal line scan Ca^2+^ imaging with DyC to selectively modulate I_tof_ and early repolarization in failing rabbit and non-failing rabbit and guinea-pig ventricular myocytes. Experimental results were simulated with a matching spatially distributed computer model for Ca^2+^ release to help clarify effects on APD morphology and Ca^2+^ release. We show that augmenting I_tof_ can increase Ca^2+^ release synchrony, Ca^2+^ transient amplitude, and cell shortening, although the effects are not monotonic and introducing too large an I_tof_ can decrease Ca^2+^ release (as seen in the aforementioned studies). Importantly, moderate I_tof_ augmentation also suppressed potentially pro-arrhythmogenic late Ca^2+^ spark activity [15] during the Ca^2+^ transient decay with minimal effects on APD.

## 2 Methods

### 2.1 Heart failure model

All procedures were carried out in accordance with the UK Home Office Animals (Scientific Procedures) Act 1986 and conformed to the guidelines from Directive 2010/63/EU of the European Parliament on the protection of animals used for scientific purposes with institutional approval from the University of Bristol ethics committee. Heart failure was induced in 3–3.5 kg New Zealand White rabbits by permanent coronary artery ligation, as described previously [15]. The established HF endpoint was <40% ejection fraction as measured by echocardiography.

### 2.2 Cardiac myocyte isolation

Rabbits were euthanised by lethal injection of 50 mg/kg sodium pentobarbital (i.v.) followed by heart removal for enzymatic cell dissociation. Left ventricular epicardial myocytes were isolated by retrograde cardiac perfusion with isolation solution (containing: 1 mg/mL collagenase I (Worthington Biochemical Corporation, NJ), 0.05 mg/mL protease (type XIV, Sigma), and 0.1 mM Ca^2+^) as described previously [17]. Ventricular myocytes were isolated from the hearts of adult male Dunkin Hartley guinea pigs using a similar enzymatic and mechanical dispersion method.

### 2.3 Electrophysiology

#### 2.3.1 General

Experiments were performed in a modified Tyrode's solution (containing, in mM: 133 NaCl, 5 KCl, 1 NaH_2_PO_4_, 10 4-(2-hydroxyethyl)-1-piperazineethanesulfonic acid (HEPES), 10 glucose, 1.8 CaCl_2_, 1 MgCl_2_, pH 7.4 with NaOH) at 36 ± 1 °C. Blebbistatin (0.01 mM) was added to the superfusate to minimise motion artefacts in Ca^2+^ records except where noted. Isoproterenol (50 nM) was added to the superfusate where indicated. APs were elicited at 1 Hz by 2 ms current injection pulses at 1.3× threshold and recordings were made after 5 consecutive beats giving near steady state behaviour with consistent cytosolic ion and SR load levels. After the 5th beat I_tof,DyC_ was added as needed and exemplar figures therefore show the immediate effect of altering I_tof_ with DyC (except when it is explicitly stated that the illustrated effects are in steady-state). Borosilicate glass patch pipettes were filled with an intracellular solution containing, in mM: 120 aspartic acid, 20 KCl, 10 HEPES, 10 NaCl, 5 glucose, 5 Mg.ATP, 0.05 Fluo-4 pentapotassium salt, with KOH added to produce pH 7.2. Pipette tip resistance was typically 1.6–2.0 MΩ with this solution. L-type Ca^2+^ currents were measured as the nifedipine-sensitive current (20 μM) in voltage clamp experiments with experimentally recorded APs as the command waveform. To inactivate Na^+^ currents, the membrane potential was reduced to −40 mV just before the upstroke of the AP. Membrane potential was recorded using an Axopatch 1D amplifier (Molecular Devices), Power1401–3 digitizer (Cambridge Electronic Design), and Signal data acquisition software (version 6.04, Cambridge Electronic Design). Cell membrane capacitance was measured by step depolarizations to −75 mV from a holding potential of −80 mV for 25 ms. Series resistance was compensated by ~70%. The liquid junction potential (10 mV) was subtracted from recordings.

#### 2.3.2 Dynamic clamping

DyC is an electrophysiological technique that employs a real-time feedback system to continuously sample V_m_ and deliver current to a cell according to a Hodgkin-Huxley model of ionic currents [18]. Changing the conductance of the injected current allows selected ionic currents to be added to the cell or, if the injected current has the opposite direction to an intrinsic current, the effect of the intrinsic current can be reduced in amplitude or nullified. This completely avoids non-specific actions of pharmacological agonists and antagonists on other cellular ionic channels/processes. Because V_m_ is free to respond to the DyC currents and excitation-contraction coupling occurs normally, the interactions between currents, cytosolic Ca^2+^ and AP morphology can be examined. This approach is distinct from the more widely-used AP voltage clamp technique [19] in which AP profile is fixed (as used in Fig. S2).

#### 2.3.3 I_tof_ formulation

In human epicardial ventricular myocytes I_tof_ is the predominant form and around ~60% of myocytes show only fast recovery from inactivation [16]. In rabbits, native I_tof_ is slower (compare rabbit data in [20] to human in [21]) and similar to the human endocardial I_tof_ (see [16]). Since human epicardial I_tof_ changes more in heart failure [21], we used the I_tof_ formulation proposed by [22] with Hodgkin-Huxley alpha/beta gating variables (Eq. (1), (2), (3), (4), (5), (6), (7)) to examine the effect of changes in a human-like I_tof_ on EC coupling by DyC:

(1) $$I_{\mathrm{tof},\mathrm{DyC}}={\bar{G}}_{\mathrm{tof},\mathrm{DyC}}X_{\mathrm{tof}\left(t\right)}Y_{\mathrm{tof}\left(t\right)}\left(V-E_{k}\right)$$

And in discrete time steps (*dt*) from *t-1* to *t*:

(2) $$X_{\mathrm{tof}\left(t\right)}=X_{\mathrm{tof}}^{\infty }-\left(X_{\mathrm{tof}}^{\infty }-X_{\mathrm{tof}\left(t-1\right)}\right)e^{-\mathrm{dt}\left(\alpha_{x}+\beta_{x}\right)}$$

(3) $$Y_{\mathrm{tof}\left(t\right)}=Y_{\mathrm{tof}}^{\infty }-\left(Y_{\mathrm{tof}}^{\infty }-Y_{\mathrm{tof}\left(t-1\right)}\right)e^{-\mathrm{dt}\left(\alpha_{y}+\beta_{y}\right)}$$

Where the steady-state (= *α*/(*α* + *β*)) and transient gating was given by the forward and backward rate constants:

(4) $$\alpha_{x}=0.04516e^{0.03577V}$$

(5) $$\beta_{x}=0.0989e^{-0.06237V}$$

(6) $$\alpha_{y}=\frac{0.005415e^{-\left(V+33.5\right)/5}}{1+0.05133e^{-\left(V+33.5\right)/5}}$$

(7) $$\beta_{y}=\frac{0.005415e^{\left(V+33.5\right)/5}}{1+0.05133e^{\left(V+33.5\right)/5}}$$

**Fig S1:**
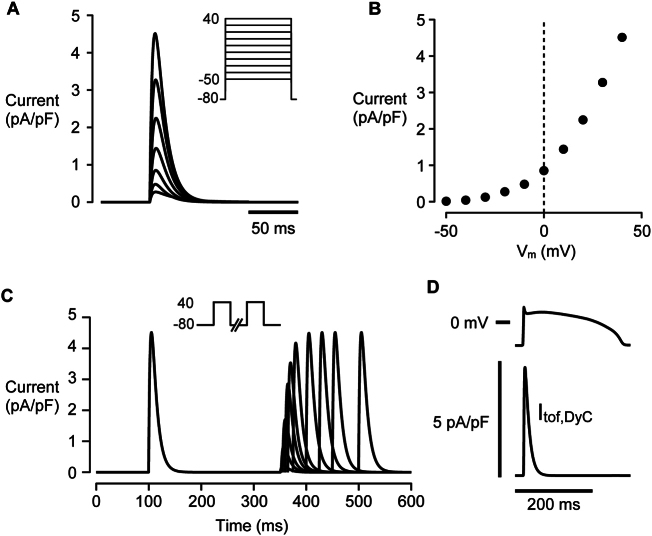
Kinetics of the mathematical model used to produce I_tof_ for DyC experiments and simulations. A I_tof_ response to steps to different holding potentials (shown inset). B Current voltage relationship for modelled I_tof_ C Recovery from inactivation. I_tof_ recovery from inactivation consisted of a single fast component and was essentially complete in 100 ms. D Time course of I_tof_ during a simulated AP.

The overall properties of the I_tof_ produced by these equations is shown in Fig. S1. Maximal conductance (${\bar{G}}_{\mathrm{tof},\mathrm{DyC}}$) was varied as described in the text and scaled by the individual cell capacitance measured at the start of each experiment. The DyC current was calculated and updated at ~10,000 times per second which is fast enough to fully recapitulate I_tof_ with minimal phase lag between instantaneous current and voltage. The same current was also varied in computer simulations of the DyC experimental data (see below) which had a background rabbit I_tof_ formulation to mimic myocyte experiments.

### 2.4 Confocal recording

Ca^2+^ sparks and transients were recorded in line scan mode from the Fluo-4 loaded cells using an inverted confocal microscope (LSM 880, Zeiss) with a 1.4 NA 63× oil immersion lens. A 488-nm argon laser provided excitation light and fluorescence emission was collected at 492–600 nm. Ca^2+^ line scan images were recorded with the pinhole set to <2 Airy units, at a pixel size of 0.1–0.2 μm/pixel, and a scan speed of 1 ms per line. GaAsP photodetectors were used to increase the sensitivity of Ca^2+^ spark detection. Late Ca^2+^ sparks (LCS) were detected using an automated Ca^2+^ spark detection algorithm in high-pass filtered recordings to suppress the low-frequency changing background fluorescence (due to the underlying Ca^2+^ transient) [17,23]. Unloaded sarcomere shortening was measured in confocal line scans along the long axis of the cell, using autocorrelation analysis of sarcomere striations imaged with the confocal transmitted light detector [24].

### 2.5 Computer model

The rabbit cardiac myocyte computer model, described in detail in Zhong et al. (2018) [25] and as modified by Hwang et al. (2020) [26], was used with additional minor modifications as described in the Online Supplement (Fig. S1).

### 2.6 Statistical analysis

Paired *t*-tests were used where data were normally distributed or logarithmically transformed to a near Gaussian distribution (assessed using Shapiro-Wilk normality test), otherwise the equivalent non-parametric test was used. Statistical analyses were performed on *n* cells from *N* rabbits, and sample sizes are presented in figure legends as *n/N*. The limit of statistical confidence was *P* < 0.05.

## 3 Results

### 3.1 Effects of I_tof_ modulation by DyC in non-failing cells

**Fig. 1:**
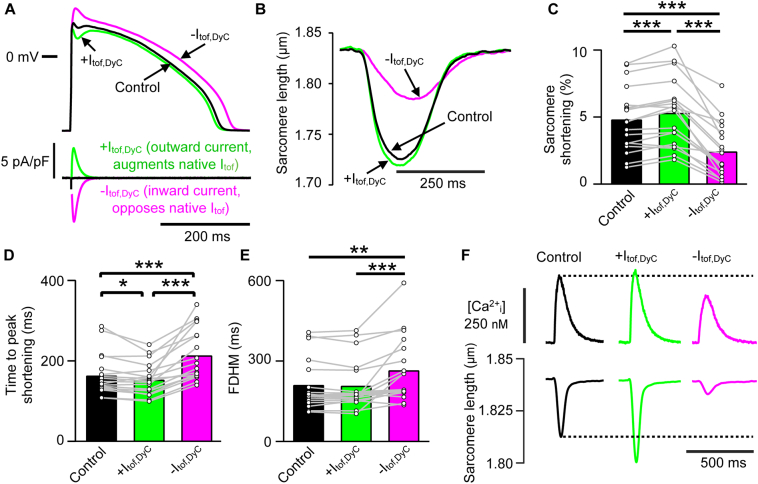
Modifying early AP repolarization with I_tof,DyC_ alters cell contraction in non-failing rabbit ventricular myocytes. A Exemplar control APs recorded from a non-failing rabbit ventricular myocyte before (black line) and after dynamic clamp with a ‘synthetic’ I_tof_ was enabled to enhance (+I_tof,DyC_, green line) or decrease (-I_tof,DyC_, magenta line) the phase 1 repolarization notch. The stimulus pulse has been partially removed for clarity. B Exemplar recording of sarcomere shortening during control APs with and without I_tof,DyC_ (no blebbistatin or Fluo-4 in pipette or extracellular solution). C + I_tof,DyC_ increased the magnitude of sarcomere shortening and (D) decreased the time to peak shortening, whereas -I_tof,DyC_ decreased shortening and prolonged the time to peak and E increased the full duration at half maximal shortening (FDHM). F Fluo-4 was included in the pipette in a subset of cells (*n* = 6) to simultaneously record intracellular Ca^2+^ during contractions. In non-failing myocytes +I_tof,DyC_ caused a modest increase in Ca^2+^ transient amplitude, whereas -I_tof,DyC_ decreased Ca^2+^ transient amplitude more severely, corresponding to an increase and decrease in contraction with +I_tof,DyC_ and -I_tof,DyC_ respectively. C-E n/*N* = 18/4, *P < 0.05, **P < 0.01, ***P < 0.001, C,D one-way repeated measures ANOVA with Tukey's post hoc test, E Friedman test with Dunn's post hoc test. (For interpretation of the references to colour in this figure legend, the reader is referred to the web version of this article.)

By calculating and injecting a time- and voltage-dependent I_tof_ with either the same (+I_tof,DyC_) or opposite polarity (-I_tof,DyC_) to the normal outward I_tof_ with DyC (see Methods), it is possible to selectively enhance or reduce the contribution of native I_tof_ to the AP without potential off-target drug effects. Fig. 1A illustrates the response of rabbit APs (control -black line) to either adding (+I_tof,DyC_ green line) or subtracting (-I_tof,DyC_ magenta line) I_tof_ as shown in the lower traces. Adding or removing I_tof_ increased or decreased the depth of the phase 1 notch respectively (confirming the ability of a synthesised I_tof_ to alter early APD time course). APD_90_ also increased slightly when the native phase 1 notch was prevented. Unloaded sarcomere shortening was measured during DyC experiments to determine whether enhancing I_tof_ had a positive or negative inotropic effect. Fig. 1B shows exemplar sarcomere length measurements during DyC experiments. +I_tof,DyC_ caused a small but significant increase in unloaded shortening and a faster time to peak shortening compared to control contractions (Fig. 1C-D) (mean shortening, control: 4.9 ± 0.6%; +I_tof,DyC_ 5.4 ± 0.6%; Time to peak, control 167 ± 12 ms, +I_tof,DyC_ 156 ± 9.6 ms, *p* < 0.05 vs control *n* = 18). In contrast, -I_tof,DyC_ caused a dramatic reduction in cell shortening, delayed the time to peak, and increased the duration of contraction (Fig. 1C-E) (mean shortening -I_tof,DyC_ 2.5 ± 0.5%; time to peak 218 ± 15 ms, *p* < 0.001 vs control). Simultaneous recordings of Ca^2+^ and contraction revealed +I_tof,DyC_ increased Ca^2+^ transient amplitude whereas -I_tof,DyC_ decreased Ca^2+^ transient amplitude in accord with the change in sarcomere shortening (Fig. 1F). These results show that enhancing I_tof_ can have a moderate inotropic effect in non-failing myocytes, whereas decreasing I_tof_ has a more obvious negative inotropic effect. This result is opposite to the reported effect of modifying I_tof_ in canine cardiomyocytes [13]. To clarify the possible cause(s) for this contradiction, further confocal line scan Ca^2+^ recordings under DyC were performed.

### 3.2 Opposing native I_tof_ impairs Ca^2+^ signalling in normal myocytes

**Fig. 2:**
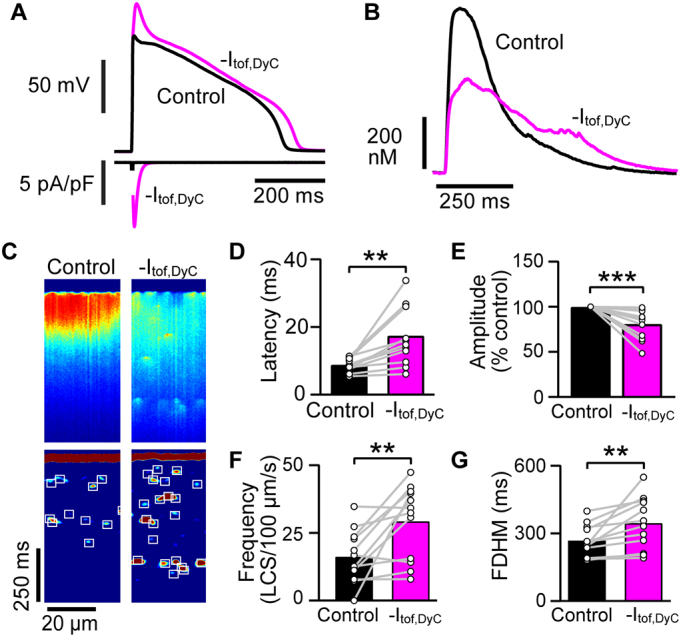
Effect of subtracting I_tof_ with DyC on AP shape and Ca^2+^ transients. A *Upper panel* Opposing native I_tof_ in non-failing rabbit myocytes using DyC abolished the small phase 1 notch and lengthened APD slightly, despite -I_tof,DyC_ fully inactivating within ~50 ms (magenta trace *lower panel*) during pacing at 1 Hz. G_tof,DyC_ was 0.1 nS/pF. The stimulus artefact has been suppressed for clarity. B -I_tof,DyC_ decreased the amplitude and increased the duration of the resulting Ca^2+^ transient. C Confocal Ca^2+^ line scan recordings reveal -I_tof,DyC_ caused a loss of Ca^2+^ release uniformity across the cell. Lower panels show processed images [17] revealing LCS activity and an approximate doubling in the number LCS frequency occurred when native I_tof_ was electrically neutralised (white boxes in lower panel). Mean results show -I_tof,DyC_ increased the latency between electrical stimulation and Ca^2+^ release (D), decreased Ca^2+^ transient amplitude (E), increased LCS frequency (F) and increased Ca^2+^ transient duration (G). n/*N* = 12/4, **P < 0.01, ***P < 0.001 vs Control. D,F,G paired *t*-test, E Wilcoxon matched-pairs signed rank test. (For interpretation of the references to colour in this figure legend, the reader is referred to the web version of this article.)

**Fig S2:**
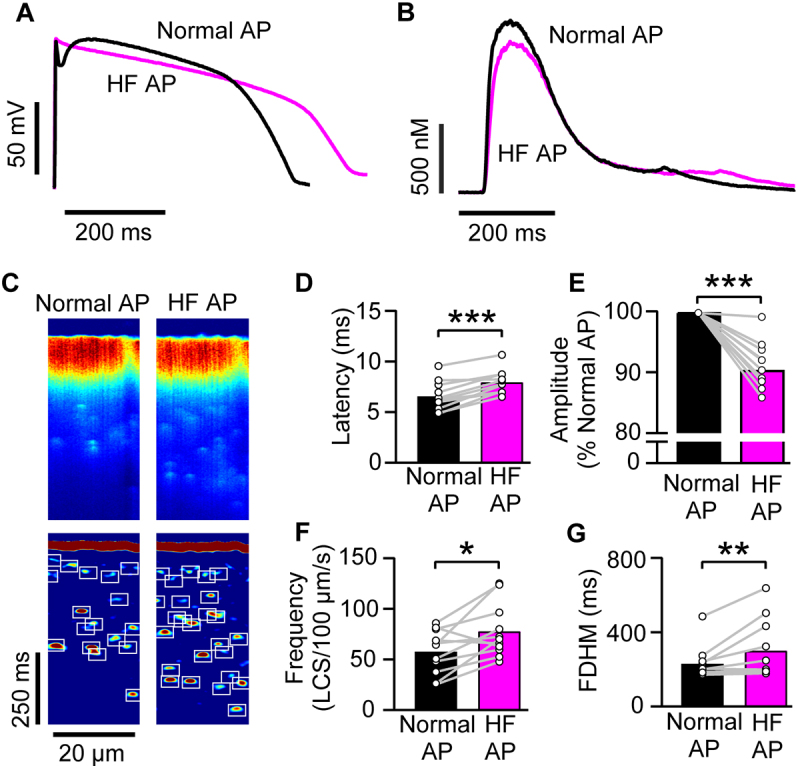
Non-failing rabbit myocytes voltage clamped with a failing AP waveform increased LCS rate. A Non-failing rabbit myocytes were voltage clamped with a train of 5 normal APs followed by a failing AP (HF) at 1 Hz to examine the role of loss of the AP notch on Ca^2+^ release and LCS. B Ca^2+^ transient amplitude was smaller and decayed more slowly with a failing AP than a normal AP. C Confocal Ca^2+^ line scans reveal greater heterogeneity in Ca^2+^ release timing with a failing AP. High-pass image processing shows many more LCS occurred in response to a failing AP (lower panels). D Average Ca^2+^ transient latency was increased, E Ca^2+^ transient amplitude decreased, and F LCS frequency and G Ca^2+^ transient duration increased with a HF AP compared to a normal AP. n/*N* = 12/4, *P < 0.05, **P < 0.01, ***P < 0.001. D,F paired *t*-test, E,G Wilcoxon matched-pairs signed rank test.

With contraction inhibited by blebbistatin (10 μmol/L), I_tof_ removal with DyC had similar effects: The Ca^2+^ transient decreased in amplitude and both the Ca^2+^ transient and AP duration increased (Fig. 2A,B) with the mean APD_90_ increasing from 315 ± 45 ms to 347 ± 51 ms (*P* = 0.012; n/*N* = 12/4). Confocal Ca^2+^ line scans (Fig. 2C upper panels) showed that -I_tof,DyC_ increased dyssynchrony in Ca^2+^ release. As recently reported [17], LCS can occur during the normal Ca^2+^ transient decay and these are shown in the lower panels in Fig. 2C (with detected LCS marked by boxes). I_tof_ removal also increased LCS frequency markedly (Fig. 2C lower panels). The changes in mean Ca^2+^ transient latency and duration, LCS frequency and Ca^2+^ transient amplitude produced by -I_tof,DyC_ are summarized in Fig. 2D-G. We also used voltage clamp with a command profile derived from measured AP time courses (also known as AP clamp [19]) to confirm these DyC results. When non-failing rabbit myocytes were voltage clamped with a failing AP lacking a phase 1 notch, Ca^2+^ transient latency, duration and LCS frequency increased and Ca^2+^ transient amplitude decreased in a similar way to the DyC results (Fig. S2).

### 3.3 Effect of adding I_tof_ in the absence of a native background I_tof_

**Fig. 3:**
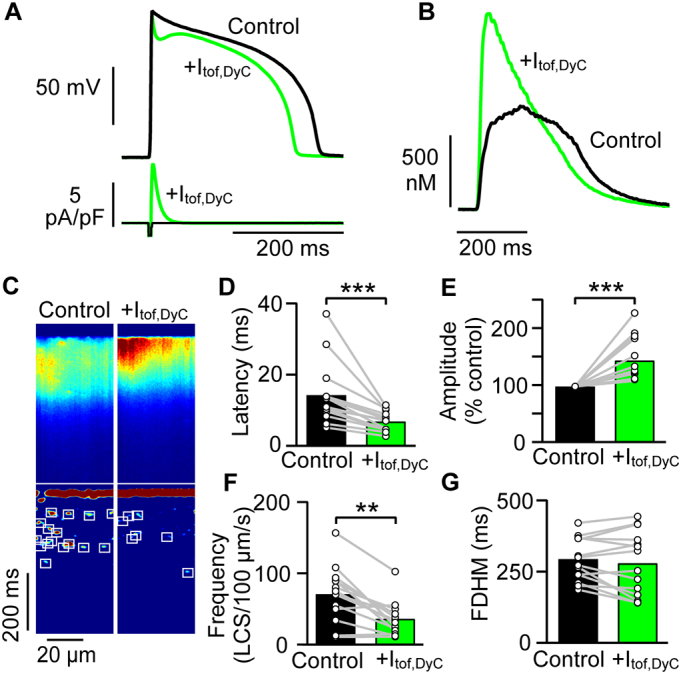
Adding I_tof,DyC_ to guinea pig myocytes synchronises Ca^2+^ release and shortens APD. A Normal guinea pig ventricular myocytes (black trace) exhibited no phase 1 notch but addition of +I_tof,DyC_ induced a phase 1 notch (green trace) reminiscent of rabbit and human APs as well as a slight shortening of APD_90_ (stimulation frequency 1 Hz). B The associated Ca^2+^ transient was larger with a faster rate of rise and decay when I_tof,DyC_ was added. C Confocal Ca^2+^ line scan recordings show some non-uniformity in Ca^2+^ release during normal Ca^2+^ transients (left panels) which was reduced by addition of I_tof,DyC_ (right panels). Lower panels show a reduction in LCS occurred with I_tof,DyC_ addition. In all cells examined (n/*N* = 14/4), +I_tof,DyC_ decreased the average Ca^2+^ transient latency (D) and increased the Ca^2+^ transient amplitude. (E) + I_tof,DyC_ decreased LCS frequency (F). +I_tof,DyC_ did not change Ca^2+^ transient duration (G; *P* = 0.32). n/N = 14/4, **P < 0.01, ***P < 0.001 vs control. D,F,G paired t-test, E Wilcoxon matched-pairs signed rank test. (For interpretation of the references to colour in this figure legend, the reader is referred to the web version of this article.)

An important consideration for interpreting whether selective I_tof_ modulation might generally improve Ca^2+^ release is the background level of I_tof_ density which, in heart failure, is reduced by the reduced expression of the molecular components of I_tof_ [3,27]. Guinea pig ventricular myocytes do not exhibit a phase 1 notch because they do not express K_v_4.2/4.3/1.4 alpha subunits [28] or a transient outward current [29], and so form a myocardial cell type with a zero I_tof_ expression background. Fig. 3A (upper panel) shows APs elicited in guinea pig ventricular myocytes under current clamp (black line). When I_tof,DyC_ with normal polarity and a magnitude similar to that seen in human left ventricular epicardial myocytes (0.1 nS/pF [16,22]) was injected using DyC, a distinct phase 1 notch appeared, consistent with previous reports [30,31] and there was an ~7% reduction in APD_90_ from 297 ± 17 ms to 278 ± 18 ms (*P* < 0.001; n/*N* = 14/4). It is notable that injecting +I_tof,DyC_ into myocytes markedly increased the amplitude of the average cellular Ca^2+^ transient from 385 ± 72 nmol/L to 586 ± 123 nmol/L (P < 0.001) and increased its rate of rise (Fig. 3B,E).

### 3.4 I_tof_ effects on Ca^2+^ release synchrony

Confocal Ca^2+^ line scan recordings (exemplar in Fig. 3C) showed both spatial and temporal heterogeneity in Ca^2+^ release site activation by the normal AP. The moderate enhancement of phase 1 repolarization by +I_tof,DyC_ markedly improved Ca^2+^ release synchrony, as quantified by the decrease in mean Ca^2+^ transient latency (Fig. 3D) and latency variance (control 7.9 ± 1.9 ms; I_tof,DyC_ 2.9 ± 0.5 ms; *P* < 0.001). Fewer LCS occurred with +I_tof,DyC_ (Fig. 3F) although Ca^2+^ transient duration did not appreciably change in the presence of +I_tof,DyC_ (*P* = 0.32; Fig. 3G). We next investigated whether enhancing I_tof_ can improve Ca^2+^ release synchrony in epicardial ventricular myocytes from a rabbit model of HF [15].

**Fig. 4:**
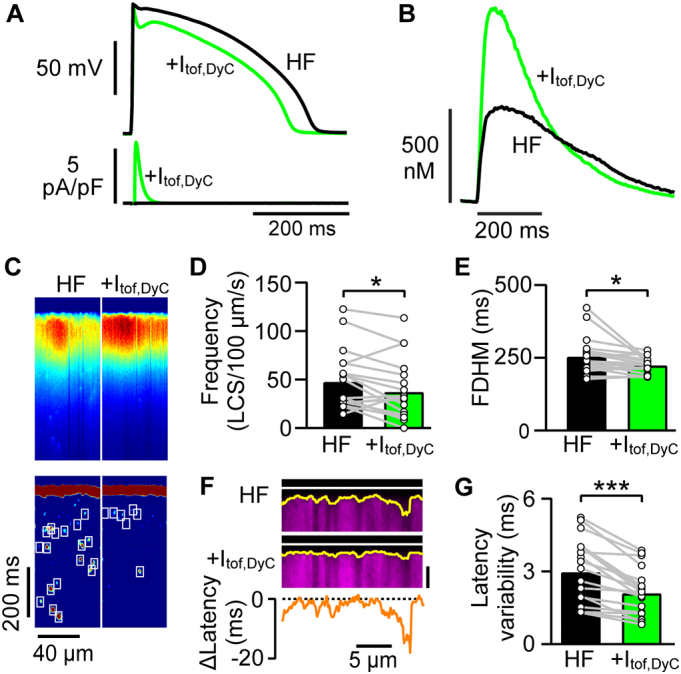
Improved Ca^2+^ release in failing cells with AP phase 1 notch restoration. A Exemplar failing rabbit ventricular myocyte (HF) AP lacking a phase 1 notch before (black) and after introducing +I_tof,DyC_ (green). +I_tof,DyC_ produced a distinct phase 1 notch and slightly shortened APD. The control APD90 in this cell was 366 ms. B The Ca^2+^ transient amplitude, rate of Ca^2+^ rise and rate of decay increased when +I_tof,DyC_ was added. C Confocal Ca^2+^ line scans show +I_tof,DyC_ increased the spatiotemporal synchrony of Ca^2+^ release throughout the cell (top panels) and decreased the number of LCS (lower panels and D) and reduced the duration of the Ca^2+^ transient (E). F Fixed patterns of Ca^2+^ release variation were detected by averaging 5 Ca^2+^ transient upstrokes before (top panel) and after adding +I_tof,DyC_ (middle panel). Ca^2+^ release latency across the cell (marked by yellow lines) following electrical stimulation (white lines). Latency (lower panel) decreased in most areas. Vertical scale bar 25 ms. G Despite regional variation, the variability in local latency was reduced by +I_tof,DyC_. n/*N* = 19/6, *P < 0.05, ***P < 0.001, D,E,G paired t-test. (For interpretation of the references to colour in this figure legend, the reader is referred to the web version of this article.)

Fig. 4A shows an exemplar AP from a failing myocyte before (black line) and after (green line) increasing I_tof_ using DyC. The +I_tof,DyC_ conductance (G_tof,DyC_) was set to 0.1 nS/pF and this produced a phase 1 notch in the failing AP and a ~ 8% shortening of APD_90_ (*P* < 0.05, n/*N* = 24/8) (from 368 ± 18 ms to 338 ± 16 ms). Adding +I_tof,DyC_ increased both the Ca^2+^ transient rate of rise and amplitude (Fig. 4B). The mean Ca^2+^ transient amplitude increased from 491 ± 34 nmol/L to 686 ± 45 nmol/L with +I_tof,DyC_ (*P* < 0.001). The exemplar line scan recordings in Fig. 4C of near steady state Ca^2+^ transients before and after adding +I_tof,DyC_ showed that the recruitment of Ca^2+^ release sites at the start of the Ca^2+^ transient increased while LCS were reduced in frequency. This reduction in LCS frequency (Fig. 4D) was also associated with a reduction in Ca^2+^ transient duration (Fig. 4E). T-tubule disruption associated with HF may limit the improvement associated with phase 1 notch restoration because uncoupled Ca^2+^ release sites should be less sensitive to AP configuration. Fig. 4F shows an exemplar line scan recording of the Ca^2+^ transient upstroke with and without +I_tof,DyC_. There was an overall reduction in Ca^2+^ release latency with +I_tof,DyC_ (HF, 10.5 ± 0.9 ms; +I_tof,DyC_, 8.7 ± 0.8 ms; P < 0.001, n/*N* = 19/6) and a small but significant reduction in latency variability (Fig. 4G). Some regions (e.g. Fig. 4F) showed a dramatic reduction in local latency. This may reflect regions in which the normal Ca^2+^ entry was marginal for triggering CICR, perhaps due to partial RyR2 uncoupling [9], or where the local increase in L-type Ca^2+^ current became sufficient to cause local Ca^2+^ release (see Discussion). It seems unlikely that the reduction in local latency could be related to local AP failure [32] as increasing I_tof_ should not improve local excitability.

### 3.5 Effects of I_tof,DyC_ on I_Ca_ and steady-state Ca^2+^ transients

**Fig. 5:**
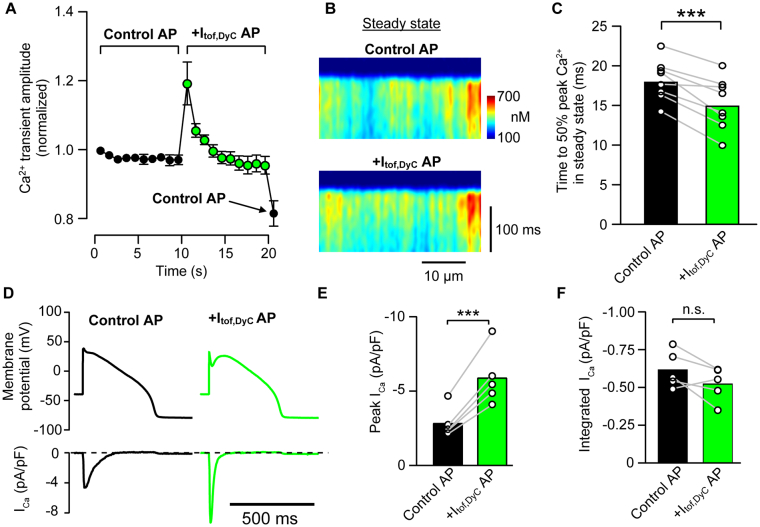
Steady state changes in I_Ca_ and Ca^2+^ release synchrony with I_tof,DyC_. A The steady state Ca^2+^ transient was established by pacing cells with a typical control AP or + I_tof,DyC_ AP as the voltage clamp command. The Ca^2+^ transient responded to a sudden change from an control AP to a + I_tof,DyC_ AP waveform with an immediate increase in amplitude. This was followed by a return to the previous steady state amplitude in 4–5 further beats. When a final control AP was reapplied (indicated by the arrow) there was a 20% reduction in the resulting Ca^2+^ transient amplitude, indicating a reduction in steady state Ca^2+^ load with +I_tof,DyC_. B Improvement in Ca^2+^ release synchrony with +I_tof,DyC_ AP persisted in steady state, as shown by a reduction in time to 50% peak Ca^2+^ release (C). D I_Ca_ measured as the nifedipine sensitive current was recorded using control and + I_tof,DyC_ AP waveforms. The membrane potential was set to −40 mV just before the AP waveform to prevent voltage escape. The +I_tof,DyC_ AP resulted in a marked increase in I_Ca_ amplitude together with a faster inactivation time course (lower panels) so that although peak I_Ca_ increased with +I_tof,DyC_ AP (E), the integral of I_Ca_ (F) was hardly different between AP waveforms (*P* > 0.05) (see also [4]). A,C n/*N* = 8/3; E,F n/*N* = 5/2; ***P < 0.001, paired t-test.

Although increasing the phase 1 notch towards 0 mV should increase peak I_Ca_ and hence SR Ca^2+^ release synchrony, increasing Ca^2+^ release without other changes can deplete the SR of Ca^2+^ [33]. When we applied the augmented I_to,f_ AP using voltage clamp, the Ca^2+^ transient amplitude increased immediately by ~20% followed by recovery to its previous steady-state amplitude during subsequent beats (Fig. 5A).

Despite there being no difference in Ca^2+^ transient amplitude at steady-state with the +I_tof,DyC_ AP, there was maintained improvement in Ca^2+^ release synchrony at steady-state (Fig. 5B,C), together with a slightly shorter Ca^2+^ transient duration (control 380 ± 31 ms; +I_tof,DyC_ 372 ± 21 ms; *P* < 0.05, n/*N* = 8/3). At the end of the pacing sequence the control AP was re-applied and this resulted in an immediate drop in Ca^2+^ transient amplitude (arrow in Fig. 5A), as might be expected if the increased SR release seen immediately upon adding I_to,f,DyC_ depleted the SR Ca^2+^ store without an increase in Ca^2+^ influx via I_Ca_ [34].

Ca^2+^ influx via I_Ca_ is sensitive to the magnitude of local SR release so that increasing release synchrony can reduce net Ca^2+^ influx due to local Ca^2+^ − dependent inactivation of Ca^2+^ channels [35]. To confirm this point, we carried out some additional experiments to measure I_Ca_ as the nifedipine-sensitive current under voltage clamp in response to the control and + I_tof,DyC_ AP waveforms described above. (Note that the resting membrane potential was reduced prior to the AP upstroke to prevent activation of I_Na_ channels and loss of voltage control). As shown in Fig. 5D,E, while peak I_Ca_ clearly increased in response to the +I_tof,DyC_ AP, it also inactivated more quickly so that the integrated Ca^2+^ influx via I_Ca_ barely changed (*P* > 0.05) (Fig. 5F). These measurements of integrated Ca^2^ influx via I_Ca_ during both APs were only slightly higher than the previously reported values for rabbit myocytes at 35 °C [36] and were not further examined (in line with 3R's goals). In summary, these experiments show that +I_tof,DyC_ was able to preserve Ca^2+^ transient amplitude despite a reduced SR Ca^2+^ content while still improving Ca^2+^ release synchrony at steady state.

### 3.6 Computer simulations of I_to,f_ effects

**Fig. 6:**
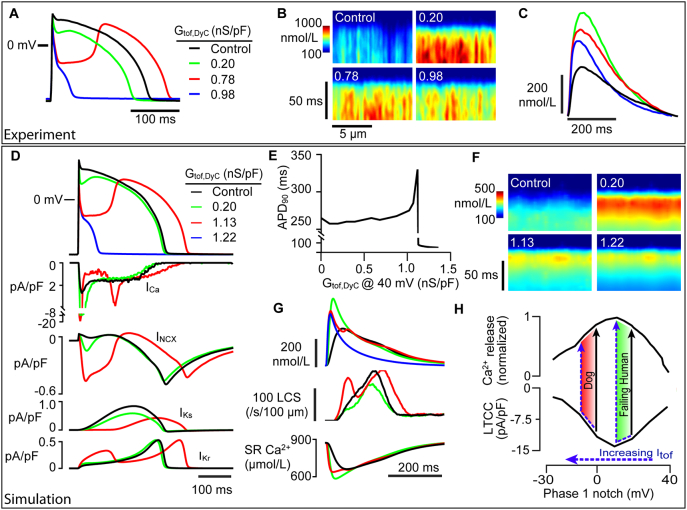
Experiments and computer simulations show a biphasic response of Ca^2+^ release and APD to increasing I_tof,DyC_. The links between Ca^2+^ release and APD_90_ with increasing G_tof,DyC_ was investigated experimentally (A-C) and with computer simulations of rabbit cardiac myocyte biophysics (D—H). A Increasing G_tof,DyC_ shifted the nadir of the AP notch to progressively more negative potentials. APD_90_ initially shortened (green trace), then lengthened (red trace), before finally collapsing to form a very short AP (blue trace). B Confocal Ca^2+^ line scans show an increase in Ca^2+^ release synchrony and (C) amplitude at ~0.2 nS/pF G_tof,DyC_ (G_tof,DyC_ values given in figure panels), followed by a decrease in amplitude and synchrony at greater G_tof,DyC_. D Simulations also showed a similar biphasic response of phase 1 notch and APD_90_ to increasing G_tof,DyC_ (top panel) with ionic currents shown in the lower panels. Moderate G_tof,DyC_ increased peak I_Ca_, decreased plateau I_Ca_ and decreased inward I_NCX_ during late repolarization (blue traces). Greater G_tof,DyC_ caused rapid termination of I_Ca_, followed by reactivation, and delayed I_Ks_ and I_Kr_ activation (green traces). I_Ca_ failed to reactivate when G_tof,DyC_ was increased to 1.22 nS/pF (not shown for clarity). E The relationship between G_tof,DyC_ and simulated APD_90_ shows a small response to increasing G_tof,DyC_, followed by a sudden increase in APD_90_ and subsequent collapse. F Simulated Ca^2+^ line scan images showed increasing G_tof,DyC_ (G_tof,DyC_ values given in figure panels) gradually decreased Ca^2+^ release latency (from 12.1 to 4.9 ms) and variability in release latency (from 3.5 to 1.1 ms) demonstrating greater Ca^2+^ release synchrony. G (top panel) Simulated Ca^2+^ transient amplitude increased with moderate G_tof,DyC_, and then declined with greater G_tof,DyC_ (colour legend same as A).LCS frequency was reduced by addition of an intermediate G_tof,DyC_ (0.2 nS/pF) and increased with higher G_tof,DyC_ (middle panel). The lower panel shows that increased early SR release at 0.2 nS/pF G_tof,DyC_ caused greater depletion of SR Ca^2+^ content. H The upper panel shows Ca^2+^ transient amplitude as a function of the corresponding AP notch nadir potential. For comparison, the lower panel shows the simulated I_Ca_ current-voltage relationship elicited by square voltage clamp steps to different holding potentials plotted on the same axis. (For interpretation of the references to colour in this figure legend, the reader is referred to the web version of this article.)

Computer models have suggested that APD_90_ has a biphasic response to increasing G_tof,DyC_ with gradual APD_90_ lengthening followed by a rapid APD_90_ shortening at a critical current density -albeit with large differences in this critical density between studies [13,30,37,38]. However, how Ca^2+^ release changes with such alterations in I_tof_ level have not been examined. Fig. 6A shows exemplar rabbit myocyte APs when G_tof,DyC_ was increased from 0.2 to 0.98 nS/pF. Increasing G_tof,DyC_ progressively drove the notch nadir V_m_ to more negative potentials and initially shortened the APD_90_, but G_tof,DyC_ > 0.2 nS/pF then lengthened the APD_90_ until an abrupt AP collapse occurred at 0.98 nS/pF. Fig. 6B shows Ca^2+^ release synchrony was enhanced by moderate G_tof,DyC_ (0.2 nS/pF) compared to the native AP. The cellular average Ca^2+^ transients in Fig. 6C also showed a biphasic response of Ca^2+^ transient amplitude with increasing G_tof,DyC_.

**Fig S3:**
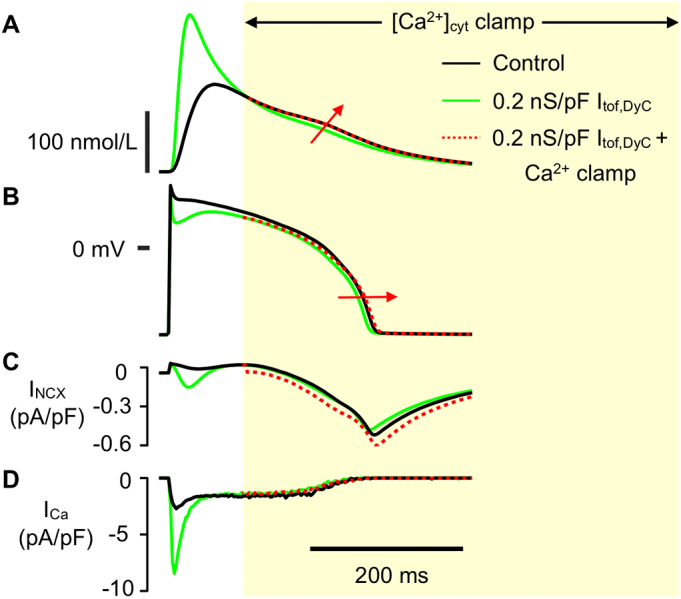
Faster Ca^2+^ transient decay contributes to action potential duration shortening with enhanced I_tof_ through reduced I_NCX_ The role of LCS and I_NCX_ in APD_90_ shortening caused by I_tof,DyC_ (0.2 nS/pF) was investigated by clamping cytosolic Ca^2+^ ([Ca^2+^]_cyt_) during computer simulations. A Traces show the simulated Ca^2+^ transient in response to the native control AP (black line) and when I_tof,DyC_ was added (green line). After the initial Ca^2+^ release and when cytosolic Ca^2+^ levels were approximately equal between simulations, the I_tof,DyC_ cytosolic Ca^2+^ was clamped to the control Ca^2+^ transient decay time course (broken red line) B Simulated APs without DyC (black line), with DyC (green line), and with DyC plus cytosolic Ca^2+^ clamp (green continuing into red dashed line). APD_90_ increases in the I_tof,DyC_ simulation when Ca^2+^ is clamped to the normal Ca^2+^ transient decay. C Inward I_NCX_ increased when Ca^2+^ was clamped to the normal Ca^2+^ transient decay during the I_tof,DyC_ simulation, likely due to increased cytosolic Ca^2+^ and slightly more negative plateau potential of the I_tof,DyC_ simulation favouring forward mode Ca^2+^ removal. D Ca^2+^ clamp had negligible effect on I_Ca_.

The cause of APD_90_ changes is complex because increased Ca^2+^ release should decrease plateau inward current via I_Ca_ (due to LTCC Ca^2+^-dependent inactivation) (Fig. S3) while at the same time moving I_NCX_ inward and it is not clear which effect should dominate. To help clarify the cause(s) of these APD_90_ changes, we used a computer model with spatially distributed Ca^2+^-release units that reproduces LCS (see Methods).

Fig. 6D shows simulated rabbit APs with normal I_tof_ density (black line), or with an additional variable I_tof,DyC_ component with a maximum conductance selected to simulate the DyC experiments shown in Fig. 6A as closely as possible (blue, green and red lines). Moderate G_tof,DyC_ caused slight APD_90_ shortening in simulated APs, whereas increasing G_tof,DyC_ further caused APD_90_ lengthening and, if increased still further, eventually led to AP collapse -as shown in experiments. The lower panels in Fig. 6D show the effect of varying G_tof,DyC_ on the main K^+^ and Ca^2+^ linked currents. Peak I_Ca_ increased as the AP notch nadir became more negative, while late plateau and early phase 3 repolarization I_Ca_, I_Ks_ and inward I_NCX_ decreased with moderate levels of G_tof,DyC_. I_Kr_ showed a more complex response to 1.13 nS/pF I_tof,DyC_ (green trace) with the deep notch potential initially promoting I_Kr_ due to reduced inactivation, then the dome phase restored a more typical I_Kr_ time course. The APD_90_ increase with larger G_tof,DyC_ is explained by I_Ca_ inactivation followed by I_Ca_ reactivation coupled with delayed and diminished I_Ks_ recruitment. Fig. 6E shows that the APD lengthens and then suddenly collapses when G_tof,DyC_ is > ~ 1.1 nS/pF in good agreement with the experimental data shown above, although somewhat higher than reported in some other simulation studies [30,37].

Simulated confocal line scans showed greater Ca^2+^ release synchrony (Fig. 6F) and increased Ca^2+^ transient amplitude with moderate G_tof,DyC_ (0.2 nS/pF) (Fig. 6G, top panel). Further increases in G_tof,DyC_ then led to a diminution of the increase in the amplitude of the Ca^2+^ transient although the increased rate of rise was maintained. The middle panel in Fig. 6G shows the time-course of appearance of LCS, which occur after a SR Ca^2+^ release refractory period (caused by the initial evoked release) [17]. Moderate G_tof,DyC_ suppressed LCS during the later stages of the Ca^2+^ transient (blue trace) compared to native I_tof_. An even larger G_tof,DyC_ caused additional LCS activity (corresponding to the upstroke of the dome) as I_Ca_ reactivated (green trace -see also Fig. 6D). To examine the extent to which LCS contributed to the APD_90_ change at 0.2 nS/pF I_tof,DyC_, we clamped the cytosolic Ca^2+^ transient decay profile to the native time course (i.e. that seen without I_tof,DyC_ addition) after the initial phase of increased Ca^2+^ release was complete (see Fig. S3). In this condition, the slowed Ca^2+^ transient decay led to a slight increase in inward I_NCX_ and APD_90_, but did not change I_Ca_. From this we conclude that a reduction in LCS due to improved early synchronous release after I_tof,DyC_ addition helps shorten APD_90_ via I_NCX_.

### 3.7 Ca^2+^ release is further improved by adding I_tof_ in the presence of β-adrenergic stimulation

**Fig. 7:**
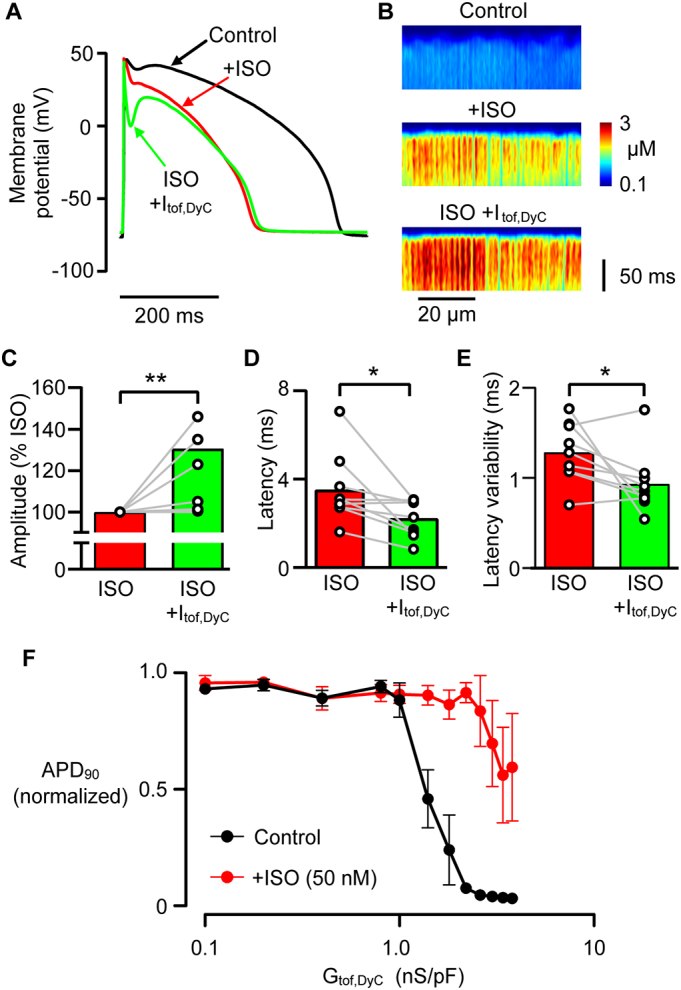
Effect of + I_tof,DyC_ on AP morphology, duration and Ca^2+^ transients in failing cells the presence of β-adrenergic stimulation. A Adding 50 nmol/l isoproterenol (ISO) shortened the AP of failing cells and slightly deepened phase 1 repolarization_._ Adding +I_tof,DyC_ (0.4 nS/pF) in the presence of ISO caused the phase 1 repolarization notch to deepen further but did not markedly change phase 2/3 or AP duration. B Line scan recordings of the Ca^2+^ transient upstroke in a failing cell in Tyrode, ISO and ISO + I_tof,DyC_. ISO improved Ca^2+^ release synchrony and amplitude which was further improved by adding +I_tof,DyC_. Compared to ISO alone, +I_tof,DyC_ (0.4 nS/pF) increased Ca^2+^ transient amplitude (C) and decreased Ca^2+^ release latency (D) and latency variability (E). Panel F shows AP duration at 90% repolarization (APD_90_) measured in response to increasing levels of +I_tof,DyC_ with and without ISO. Data are normalized to the control APD_90_ (G_tof,DyC_ = 0 nS/pF) in Tyrode's or ISO solutions. APD_90_ in Tyrode decreased sharply when +I_tof,DyC_ was increased beyond a threshold conductance (~1nS/pF). In ISO this threshold value was increased to >2 nS/pF. n/*N* = 9/3, *P < 0.05, **P < 0.01 vs ISO, C Wilcoxon matched-pairs signed rank test, D,E paired t-test.

In heart failure, impaired excitation-contraction coupling (ECC) may be partially offset by β-adrenergic stimulation of I_Ca_ and increased SR load [39] that can improve SR release synchrony [5,11,40]. As might be expected, 50 nmol/L isoproterenol (ISO) shortened the action potential and increased the amplitude of the Ca^2+^ transient (Fig. 7A, B). ISO also significantly reduced the overall latency for Ca^2+^ release to 3.6 ± 0.5 ms (*P* < 0.001; unpaired *t*-test) and the variability in local SR release to 1.3 ± 0.1 ms (P < 0.001; unpaired t-test). It is notable that applying +I_tof,DyC_ (G_tof,DyC_ = 0.4 nS/pF) in these conditions further improved the spatio-temporal properties of the Ca^2+^ transient by increasing the amplitude and reducing the latency and dispersion of Ca^2+^ release (Fig. 7C,D,E). This shows that increasing the depth of the phase 1 notch towards 0 mV further improves ECC synchrony in failing cells even if ECC is already enhanced by β-adrenergic stimulation [39]. During these experiments, we also noticed that while the APD_90_ responded similarly to increasing +I_tof,DyC_ in failing cells as in non-failing cells in Tyrode solution (with abrupt AP collapse developing around 1 nS/pF (n/*N* = 12/3; Fig. 7F), isoproterenol allowed a larger I_tof_ to be applied (up to ~2 nS/pF) before sudden AP shortening appeared.

## 4 Discussion

The experiments reported here illustrate the complex interplay between early Ca^2+^ release, LCS and APD that is modulated by I_tof_. In both guinea pig (where I_tof_ is absent) and rabbit, where phase 1 is more akin to the reduced phase 1 seen in human heart failure, adding physiological levels of I_tof_ improved synchrony in myocyte Ca^2+^ release. Additional effects on APD depend on the amount of I_tof_ introduced and we note that a moderate reduction in APD might help normalize the prolongation of the failing APD, although the possible benefit of this effect will require additional studies.

### 4.1 Consequences of I_tof_ removal

The increase in APD_90_ when I_tof_ was removed using DyC (Fig. 1A, Fig. 2A) can be explained by an increase in plateau I_Ca_ and inward I_NCX._ The former can be explained by a reduction in SR Ca^2+^ release-dependent inhibition of I_Ca_ [35] while the latter arises from an increase in LCS frequency due to decreased SR depletion following the decrease in earlier synchronous release [17]. This APD_90_ increase is consistent with results obtained by pharmacological inhibition of I_tof_ in right ventricular epicardial myocytes [41]. With regard to regional variation in I_tof_, it is important to note that I_tof_ density is greater in canine right ventricle than left ventricle [42] and greater in the left ventricle epicardium than endocardium [43]. Since the driving force for Ca^2+^ entry via LTCC increases with phase 1 notch depth, this regional variation of I_tof_ may be offset by regional variations in I_Ca_ density to preserve the overall LTCC Ca^2+^ influx (trigger for SR Ca^2+^ release), SR loading and Ca^2+^ transient amplitude [44]. In connection with this, we note that guinea pig myocytes function perfectly well without a phase 1 notch but may well compensate with a larger I_Ca_ density of up to ~22 pA/pF at +10 mV [35].

### 4.2 Consequences of I_tof_ augmentation

Increasing G_tof,DyC_ monotonically increased the depth of notch, but APD_90_ and Ca^2+^ release properties exhibited more complex behaviour. Moderate facilitation of I_tof_ significantly synchronised Ca^2+^ release and suppressed LCS, while causing a slight reduction in APD_90_ in both experiments and simulations. The simplest explanation for the APD_90_ reduction with moderate facilitation of I_tof_ is that more synchronous SR release causes an increase in Ca^2+^-dependent inactivation of I_Ca_ (as seen in Fig. 5D), as well as a reduction in inward I_NCX_ during phase 3 (due to a reduction in LCS contributing to a shorter Ca^2+^ transient) which was sufficient to overcome a slight reduction in I_Ks_ following the change in early AP time course (Fig. 6D).

It was notable then when we added ~6 pA/pF I_tof_ to the intrinsic ~4pA/pF rabbit I_to_, the resultant AP waveform became very similar to human epicardial AP waveforms (see e.g. Fig. 1 in [21]) where the I_tof_ density is ~10 pA/pF [21,45]. This observation leads us to suggest that our rabbit results may well predict how human epicardial myocytes would respond to I_tof_ modulation. We recently showed how LCS (whose rate is increased by dyssynchronous early release) can translate to an increase in the risk for EAD's [15]. Therefore, in heart failure, increasing I_tof_ could potentially reduce the risk for arrhythmia development and subsequent sudden cardiac death. In connection with this, the ability to increase I_tof_ without AP collapse is an important consideration.

### 4.3 Comparison with other studies examining I_to,f_ modulation

While no APD_90_ shortening was observed in previous DyC and computer modelling studies in canine and guinea pig ventricular myocytes [30,37,38], it is difficult to compare such results to those presented here without simultaneous Ca^2+^ measurements (as Ca^2+^ affects ionic currents during repolarization). Adenoviral transduction of K_v_4.3 into guinea pig hearts shortened the APD but these results are complicated by the lack of channel beta-subunits necessary to produce the fast activation and recovery kinetics of the native current [46]. However, we recently reported that adenoviral transduction of rabbit cardiac myocytes with both Kv4.3 and KChip2.1 can recapitulate I_to,f_ [47] and these experiments showed that the AP did not collapse until I_to,f_ was ~12 pA/pF at +40 mV, in good agreement with the results presented here. Nevertheless, our experimental results, supported by computer simulations, show Ca^2+^ release synchrony in heart failure can be improved by adding I_tof_, with only small changes in APD_90_.

In cells with larger I_tof_ densities (such as canine RV and LV epicardium), increasing outward currents at the end of phase 1 can decrease the activation of I_Ca_ [37], causing heterogeneous loss of the AP dome and increased transmural repolarization dispersion. In connection with this, a Brugada-like syndrome was induced in canine ventricular wedge preparations perfused with the I_tof_ agonist NS5806 [48], although this result may have been influenced by the wedge preparation methodology itself [49]. However in rabbit, NS5806 did not cause Brugada-like behaviour although Ca^2+^ and V_m_ alternans [50] appeared at high concentrations (45 μM) where I_Na_ is also inhibited [20,48]. It is also possible that a part of this species-dependent behaviour is related to the I_Ca_ density which is reported to be smaller in dog ventricular myocytes (~2.5–4 pA/pF at 0 mV [44,51]) than in rabbit and human myocytes (~10 pA/pF as seen in the present study and [52]) and a smaller I_Ca_ density may make the AP plateau more prone to collapse because, as is well known, I_Ca_ is key to sustaining the AP plateau. Since we were able to increase I_tof_ without a steady state reduction in Ca^2+^ transient amplitude, our data suggests that I_tof_ may not be a negative regulator of contractility as previously suggested for canine preparations [13].

The presence of a positive notch potential in rabbit and human HF myocytes allows a graded improvement in Ca^2+^ release synchrony, because an increase in I_tof_ in these cell types will move the notch nadir towards 0 mV and increase I_Ca_ due to the bell-shaped I_Ca_ -V_m_ relationship (see Fig. 6H) which then increases CICR [53] (see also Fig. 3 in [4]), thereby improving the synchrony of Ca^2+^ release and rate of rise of the Ca^2+^ transient. In the steady state, increasing peak I_Ca_ may also increase SR release synchrony -even if SR Ca^2+^ levels are relatively unaffected (see Fig. 5 and [54]). The decrease in SR load that we observed during steady state pacing with a typical I_tof,DyC_ waveform could provide some protection against diastolic Ca^2+^ sparks and delayed afterdepolarizations. However, if I_tof_ facilitation shifts the phase 1 nadir below the peak of the I_Ca_-V_m_ relationship (which occurs at about +10 mV) [55] this could reduce I_Ca_. In connection with this point, we note that canine epicardial myocytes have a deep phase 1 notch potential to between −12 mV and − 10 mV [56] which is clearly in the range where any further increase in notch depth by I_to,f_ augmentation will decrease I_Ca_ and hence SR Ca^2+^ release [13,53] (see shaded bars in Fig. 5H).

Loss of I_tof_ will also reduce the rate of phase 1 repolarization which has also been shown to affect the synchrony of Ca^2+^ release via effects on I_Ca_ time course [5]. It follows that actual degree of SR Ca^2+^ dyssynchrony reflects a complex interplay between AP repolarization rate and voltage during phase 1. However, it is clear that a rapid, more spike-like AP and I_Ca_ is beneficial to synchronize Ca^2+^ release [57]. Although Ca^2+^ release synchrony naturally increases with isoproterenol [5,40] (due to increased I_Ca_ and SR Ca^2+^ content), adding I_tof_ further improved Ca^2+^ release synchrony. This beneficial effect is underscored by our observation that in steady state, the amplitude of the Ca^2+^ transient was hardly altered (Fig. 5A) due to autoregulation [54] while the improvement in Ca^2+^ release synchrony (and increased rate of Ca^2+^ transient decay) persisted. Thus the steady-state effects of I_tof_ augmentation may not be “inotropic” in the common physiological sense (which generally describes “inotropy” in terms of the magnitude of force (P) development) the decrease in time to 50% peak Ca^2+^ (Fig. 5C) may translate to some improvement in dP/dt since there is a monotonic relationship between instantaneous Ca^2+^ and dF/dt in intact cardiac muscle [58]. In heart failure, both the rate of isovolumic pressure development and pressure decay are correlated with clinical outcome [59] so that the changes in the rate of rise and decline of the Ca^2+^ transient, as well as the suppression of LCS, that we have observed may be beneficial.

### 4.4 Could I_to,f_ augmentation be used therapeutically?

While selective enhancement of I_tof_ has the potential to be therapeutic in the context of heart failure, the APD shortening seen at high levels of I_tof_ may be problematic and this needs further study. However, in our experiments, adding a β-agonist increased the APD collapse threshold (Fig. 7F) and could broaden the therapeutic range for I_tof_ enhancement (although β-adrenergic desensitization and/or downregulation [60] may limit this effect). On the other hand, any AP shortening effect could help normalize AP duration in the context of heart failure or some long QT syndromes. Transmural differences in I_tof_ density [43] may need to be preserved to prevent abnormal repolarization gradients forming, so future development of these ideas may have to avoid simply increasing I_tof_ uniformly across the wall but instead seek to reproduce (or even enhance) the natural transmural I_tof_ gradient.

## 5 Conclusions and limitations for translation

Augmenting I_tof_ in failing ventricular myocytes increases phase 1 and, via consequent effects on I_Ca_, can synchronize Ca^2+^ release while suppressing LCS during the Ca^2+^ transient decline. These improvements in Ca^2+^ handling can occur with only small effects on APD_90_. However, increasing I_tof_ above physiological levels may be problematic due to the potential for AP collapse, although it is uncertain whether this (undesirable) effect would occur at therapeutic levels of I_tof_ augmentation in humans. It is also possible that such a side effect of I_tof_ augmentation may be controllable by certain anti-arrhythmic drugs and future studies should also examine this possibility. Nevertheless, these data show that restoration of phase 1 repolarization via I_tof_ is a potential route towards improving cardiac Ca^2+^ release synchrony and reducing the risk of sudden cardiac death in heart failure. While the dynamic clamp method used here is not a viable approach for translation, our data suggests that I_tof_ restoration via drugs or transgene expression should be examined further.

The following are the supplementary data related to this article.

Supplementary material: Expanded Materials & Methods Supplementary material

## Sources of funding

The authors acknowledge funding from 10.13039/501100000265Medical Research Council UK Program Grant MR/N002903/1 and 10.13039/501100000274British Heart Foundation (PG/15/106/31915).

## Disclosures

None.

## Data availability

The data underlying this article will be shared on reasonable request to the corresponding author.

## References

[1] Wettwer E., Amos G., Gath J., Zerkowski H.R., Reidemeister J.C., Ravens U. (1993). Transient outward current in human and rat ventricular myocytes. Cardiovasc. Res..

[2] Näbauer M., Beuckelmann D.J., Erdmann E. (1993). Characteristics of transient outward current in human ventricular myocytes from patients with terminal heart-failure. Circ. Res..

[3] Kääb S., Dixon J., Duc J., Ashen D., Näbauer M., Beuckelmann D.J. (1998). Molecular basis of transient outward potassium current downregulation in human heart failure - A decrease in Kv4.3 mRNA correlates with a reduction in current density. Circulation..

[4] Cooper P.J., Soeller C., Cannell M.B. (2010). Excitation-contraction coupling in human heart failure examined by action potential clamp in rat cardiac myocytes. J. Mol. Cell. Cardiol..

[5] Sah R., Ramirez R.J., Backx P.H. (2002). Modulation of Ca2+ release in cardiac myocytes by changes in repolarization rate. Circ. Res..

[6] Sah R., Ramirez R.J., Oudit G.Y., Gidrewicz D., Trivieri M.G., Zobel C. (2002). Regulation of cardiac excitation-contraction coupling by action potential repolarization: role of the transient outward potassium current (Ito). J. Physiol. Lond..

[7] Harris D.M., Mills G.D., Chen X., Kubo H., Berretta R.M., Votaw V.S. (2005). Alterations in early action potential repolarization causes localized failure of sarcoplasmic reticulum Ca2+ release. Circ. Res..

[8] Crossman D.J., Young A.A., Ruygrok P.N., Nason G.P., Baddelely D., Soeller C. (2015). T-tubule disease: Relationship between t-tubule organization and regional contractile performance in human dilated cardiomyopathy. J. Mol. Cell. Cardiol..

[9] Song L.S., Sobie E.A., McCulle S., Lederer W.J., Balke C.W., Cheng H.P. (2006). Orphaned ryanodine receptors in the failing heart. Proc. Natl. Acad. Sci..

[10] Konstam M.A., Kramer D.G., Patel A.R., Maron M.S., Udelson J.E. (2011). Left ventricular remodeling in heart failure: current concepts in clinical significance and assessment. JACC Cardiovasc. Imaging.

[11] Heinzel F.R., MacQuaide N., Biesmans L., Sipido K. (2011). Dyssynchrony of Ca2+ release from the sarcoplasmic reticulum as subcellular mechanism of cardiac contractile dysfunction. J. Mol. Cell. Cardiol..

[12] Cordeiro J.M., Calloe K., Moise N., Kornreich B., Giannandrea D., Di Diego J.M. (2012). Physiological consequences of transient outward K+ current activation during heart failure in the canine left ventricle. J. Mol. Cell. Cardiol..

[13] Dong M., Yan S., Chen Y., Niklewski P.J., Sun X., Chenault K. (2010). Role of the transient outward current in regulating mechanical properties of canine ventricular myocytes. J. Cardiovasc. Electrophysiol..

[14] Maleckar M.M., Lines G.T., Koivumäki J.T., Cordeiro J.M., Calloe K. (2014). NS5806 partially restores action potential duration but fails to ameliorate calcium transient dysfunction in a computational model of canine heart failure. Europace..

[15] Fowler E.D., Wang N., Hezzell M., Chanoit G., Hancox J.C., Cannell M.B. (2020). Arrhythmogenic late Ca2+ sparks in failing heart cells and their control by action potential configuration. Proc. Natl. Acad. Sci..

[16] Johnson E.K., Springer S.J., Wang W., Dranoff E.J., Zhang Y., Kanter E.M. (2018). Differential expression and remodeling of transient outward potassium currents in human left ventricles. Circ. Arrhythm. Electrophysiol..

[17] Fowler E.D., Kong C.H.T., Hancox J.C., Cannell M.B. (2018). Late Ca2+ sparks and ripples during the systolic Ca2+ transient in heart muscle cells. Circ. Res..

[18] Sharp A.A., O'Neil M.B., Abbott L.F., Marder E. (1993). Dynamic clamp: computer-generated conductances in real neurons. J. Neurophysiol..

[19] Doerr T., Denger R., Doerr A., Trautwein W. (1990). Ionic currents contributing to the action-potential in single ventricular myocytes of the guinea-pig studied with action-potential clamp. Pflugers Arch..

[20] Cheng H., Cannell M.B., Hancox J.C. (2017). Differential responses of rabbit ventricular and atrial transient outward current (Ito) to the Ito modulator NS5806. Phys. Rep..

[21] Näbauer M., Beuckelmann D.J., Uberfuhr P., Steinbeck G. (1996). Regional differences in current density and rate-dependent properties of the transient outward current in subepicardial and subendocardial myocytes of human left ventricle. Circulation..

[22] Winslow R.L., Rice J., Jafri S., Marbán E., O'Rourke B. (1999). Mechanisms of altered excitation-contraction coupling in canine tachycardia-induced heart failure, II - Model studies. Circ. Res..

[23] Kong C.H.T., Soeller C., Cannell M.B. (2008). Increasing sensitivity of Ca2+ Spark detection in noisy images by application of a matched-filter object detection algorithm☆. Biophys. J..

[24] Peterson P., Kalda M., Vendelin M. (2013). Real-time determination of sarcomere length of a single cardiomyocyte during contraction. AJP: Cell Physiology..

[25] Zhong M., Rees C.M., Terentyev D., Choi B.-R., Koren G., Karma A. (2018). NCX-mediated subcellular Ca2+ dynamics underlying early afterdepolarizations in LQT2 cardiomyocytes. Biophys. J..

[26] Hwang J., Kim T.Y., Terentyev D., Zhong M., Kabakov A.Y., Bronk P. (2020). Late INa Blocker GS967 supresses polymorphic ventricular tachycardia in a transgenic rabbit model of long QT Type 2. Circ. Arrhythm. Electrophysiol..

[27] Radicke S., Cotella D., Graf E., Banse U., Jost N., Varro A. (2006). Functional modulation of the transient outward current Ito by KCNE β-subunits and regional distribution in human non-failing and failing hearts. Cardiovasc. Res..

[28] Zicha S., Moss I., Allen B., Varró A., Papp J., Dumaine R. (2003). Molecular basis of species-specific expression of repolarizing K+ currents in the heart. Am. J. Physiol. Heart Circ. Physiol..

[29] Findlay I. (2003). Is there an A-type K+ current in guinea pig ventricular myocytes?. Am. J. Physiol. Heart Circ. Physiol..

[30] Dong M., Sun X., Prinz A.A., Wang H.S. (2006). Effect of simulated I(to) on guinea pig and canine ventricular action potential morphology. Am. J. Physiol. Heart Circ. Physiol..

[31] Sala L., Hegyi B., Bartolucci C., Altomare C., Rocchetti M., Váczi K. (2018). Action potential contour contributes to species differences in repolarization response to β-adrenergic stimulation. Europace..

[32] Sacconi L., Ferrantini C., Lotti J., Coppini R., Yan P., Loew L.M. (2012). Action potential propagation in transverse-axial tubular system is impaired in heart failure. Proc. Natl. Acad. Sci..

[33] Eisner D. (2014). Calcium in the heart: from physiology to disease. Exp. Physiol..

[34] Dibb K.M., Graham H.K., Venetucci L.A., Eisner D.A., Trafford A.W. (2007). Analysis of cellular calcium fluxes in cardiac muscle to understand calcium homeostasis in the heart. Cell Calcium.

[35] Grantham C.J., Cannell M.B. (1996). Ca2+ influx during the cardiac action potential in guinea pig ventricular myocytes. Circ. Res..

[36] Puglisi J.L., Yuan W., Bassani J.W., Bers D.M. (1999). Ca(2+) influx through Ca(2+) channels in rabbit ventricular myocytes during action potential clamp: influence of temperature. Circ. Res..

[37] Greenstein J.L., Wu R., Po S., Tomaselli G.F., Winslow R.L. (2000). Role of the calcium-independent transient outward current I(to1) in shaping action potential morphology and duration. Circ. Res..

[38] Sun X., Wang H.-S. (2005). Role of the transient outward current (Ito) in shaping canine ventricular action potential--a dynamic clamp study. J. Physiol. Lond..

[39] Gomez A.M., Valdivia H.H., Cheng H., Lederer M.R., Santana L.F., Cannell M.B. (1997). Defective excitation-contraction coupling in experimental cardiac hypertrophy and heart failure. Science..

[40] Litwin S.E., Zhang D., Bridge J.H. (2000). Dyssynchronous Ca(2+) sparks in myocytes from infarcted hearts. Circ. Res..

[41] Virág L., Jost N., Papp R., Koncz I., Kristóf A., Kohajda Z. (2011). Analysis of the contribution of I(to) to repolarization in canine ventricular myocardium. Brit. J. Pharmacol..

[42] Volders P.G., Sipido K.R., Carmeliet E., Spätjens R.L., Wellens H.J., Vos M.A. (1999). Repolarizing K+ currents ITO1 and IKs are larger in right than left canine ventricular midmyocardium. Circulation..

[43] Zicha S., Xiao L., Stafford S., Cha T.J., Han W., Varró A. (2004). Transmural expression of transient outward potassium current subunits in normal and failing canine and human hearts. J. Physiol. Lond..

[44] Cordeiro J.M., Greene L., Heilmann C., Antzelevitch D., Antzelevitch C. (2004). Transmural heterogeneity of calcium activity and mechanical function in the canine left ventricle. Am. J. Physiol. Heart Circ. Physiol..

[45] Akar F.G. (2004). Phenotypic differences in transient outward K+ current of human and canine ventricular myocytes: insights into molecular composition of ventricular Ito. Am. J. Physiol. Heart Circ. Physiol..

[46] Hoppe U.C., Marbán E., Johns D.C. (2000). Molecular dissection of cardiac repolarization by in vivo Kv4.3 gene transfer. J. Clin. Invest..

[47] Wang N., Dries E., Fowler E.D., Harmer S.C., Hancox J.C., Cannell M.B. (2022). Inducing Ito,f and phase 1 repolarization of the cardiac action potential with a Kv4.3/KChIP2.1 bicistronic transgene. J. Mol. Cell. Cardiol..

[48] Calloe K., Cordeiro J.M., Di Diego J.M., Hansen R.S., Grunnet M., Olesen S.-P. (2009). A transient outward potassium current activator recapitulates the electrocardiographic manifestations of Brugada syndrome. Cardiovasc. Res..

[49] Boukens B.J., Meijborg V.M.F., Belterman C.N., Opthof T., Janse M.J., Schuessler R.B. (2017). Local transmural action potential gradients are absent in the isolated, intact dog heart but present in the corresponding coronary-perfused wedge. Phys. Rep..

[50] Wang S., Rodríguez-Mañero M., Ibarra-Cortez S.H., Kreidieh B., Valderrábano L., Hemam M. (2019). NS5806 induces electromechanically discordant alternans and arrhythmogenic voltage-calcium dynamics in the isolated intact rabbit heart. Front. Physiol..

[51] Wang H.-S., Cohen I.S. (2003). Calcium channel heterogeneity in canine left ventricular myocytes. J. Physiol. Lond..

[52] Piacentino V., Weber C.R., Chen X., Weisser-Thomas J., Margulies K.B., Bers D.M. (2003). Cellular basis of abnormal calcium transients of failing human ventricular myocytes. Circ. Res..

[53] Cannell M.B., Berlin J.R., Lederer W.J. (1987). Effect of membrane-potential changes on the calcium transient in single-rat cardiac-muscle-cells. Science..

[54] Diaz M.E., Graham H.K., O'Neill S.C., Trafford A.W., Eisner D.A. (2005). The control of sarcoplasmic reticulum Ca content in cardiac muscle. Cell Calcium.

[55] Rubart M., Lopshire J.C., Fineberg N.S., Zipes D.P. (2000). Changes in left ventricular repolarization and ion channel currents following a transient rate increase superimposed on bradycardia in anesthetized dogs. J. Cardiovasc. Electrophysiol..

[56] Dong M., Niklewski P.J., Wang H.-S. (2011). Ionic mechanisms of cellular electrical and mechanical abnormalities in Brugada syndrome. Am. J. Physiol. Heart Circ. Physiol..

[57] Bridge J.H., Ershler P.R., Cannell M.B. (1999). Properties of Ca2+ sparks evoked by action potentials in mouse ventricular myocytes. J. Physiol. Lond..

[58] Yue D.T. (1987). Intracellular [Ca2+] related to rate of force development in twitch contraction of heart. Am. J. Phys..

[59] Kolias T.J., Aaronson K.D., Armstrong W.F. (2000). Doppler-derived dP/dt and -dP/dt predict survival in congestive heart failure. Jacc..

[60] Lymperopoulos A., Rengo G., Koch W.J. (2013). Adrenergic nervous system in heart failure: pathophysiology and therapy. Circ. Res..

