# Supplementary material for: Improved Ca2+ release synchrony following selective modification of Itof and phase 1 repolarization in normal and failing ventricular myocytes
Source: J Mol Cell Cardiol. 2022 Nov;172:52–62. doi: 10.1016/j.yjmcc.2022.07.009 (PMC11773631; doi:10.1016/j.yjmcc.2022.07.009)
Supplement: Supplementary material: Expanded Materials & Methods [file mmc4.docx]

**Supplemental Materials**

Expanded Materials & Methods

*Computer model*

We used a detailed rabbit ventricular myocyte computer model, originally based on that of Restrepo et al. with modification by Terentyev et al. (see MS) To replicate experimental conditions, simulated APs were evoked at a cycle length of 1000 ms to standardise SR loading then DyC was enabled. Native I_to_ in the model was replaced with a modified formalism described in Choi *et al.* (2018) [1] as the sum of fast inactivating/recovering and slowly inactivating/recovering components and updated with our experimental data of I_to_ in rabbit:

|  | $I_{to}= \bar{G}_{tof}X_{to(t)}Y_{tof(t)}\left( V-E_{k} \right)+ \bar{G}_{tos}X_{to(t)}Y_{tos(t)}(V-E_{k})$ | Eq 1 |
| --- | --- | --- |
|  | $\bar{G}_{tof}= 0.0295 nS/pF$ |  |
|  | $\bar{G}_{tos}= 0.0295 nS/pF$ |  |
|  | $X_{to}^{\infty}= \frac{1}{1+e^{-(V+12.17)/11.59}}$ | Eq 2 |
|  | $Y_{to}^{\infty}= \frac{1-R}{1+e^{(V+40.9)/12}}+R$ | Eq 3 |
|  | $\tau_{Xto}= \frac{9}{1+e^{(V+3)/15}}+0.5$ | Eq 4 |
|  | $\tau_{Ytof}= \frac{100}{1+e^{(V+60)/10}}+8.4$ | Eq 5 |
|  | $\tau_{Ytos}= \frac{3000}{1+e^{(V+60)/10}}+50$ | Eq 6 |
|  | $X_{to(t)}= X_{to}^{\infty}-\left( X_{to}^{\infty}- X_{to(t-1)} \right)e^{-dt/\tau_{Xto}}$ | Eq 7 |
|  | $Y_{tof(t)}= Y_{to}^{\infty}-\left( Y_{to}^{\infty}- Y_{tof(t-1)} \right)e^{-dt/\tau_{Ytof}}$ | Eq 8 |
|  | $Y_{tos(t)}= Y_{to}^{\infty}-\left( Y_{to}^{\infty}- Y_{tos(t-1)} \right)e^{-dt/\tau_{Ytos}}$ | Eq 9 |

Where /G_tof_ and /G_tos_ are the maximal conductance of fast and slow components. The same steady state activation ($X_{to}^{\infty}$) and inactivation ($Y_{to}^{\infty}$) parameters were used for fast and slow components. A non-inactivating component, R = 0.1, was included to account for voltage-dependent steady state current at the end of 300 ms voltage clamp holding steps. X_to_, Y_tof_ and Y_tos_ are the instantaneous activation and inactivation parameters. To simulate experiments, the formalism for the experimental I_tof,DyC_ (see MS) was added to the model equations as required. Other model parameters adjusted from Hwang et al. (2020) [2] are shown in Table S1. I_Ca_ conductance was adjusted to yield a similar peak current during simulated I-V as measured in non-failing and failing rabbit ventricular myocytes. The rate of LTCC Ca^2+^-dependent inactivation and recovery from inactivation and the voltage dependence of steady state inactivation were adjusted as detailed in Table S1. Time-dependent allosteric NCX activation was implemented as described in Zhong et al. (2018) [3]. Ca^2+^ release events were categorised as LCS if they produced sufficient RyR2 release flux (>3000 µmol Ca^2+^/ms) and occurred after a refractory period following evoked Ca^2+^ release. LCS refractory period is dependent on SR refilling and at 24°C recovers with a half-time of 119 ms. The LCS refractory period was reduced to account for faster SERCA2a activity at 37°C (SERCA2a Q_10_ ~2.6). Simulated confocal Ca^2+^ line scans were constructed by convolving spatiotemporal cytosolic Ca^2+^ in the centre of the model cell with an ideal point-spread function of confocal microscope optics.

| Model parameter | Value |
| --- | --- |
| G_Ks_ | 0.20 nS/pF |
| G_Kr_ | 0.02 nS/pF |
| G_Na,L_ | 0.0025 nS/pF |
| G_NaK_ | 1.50 nS/pF |
| Transverse Ca^2+^ diffusion time | 0.33 ms |
| Longitudinal Ca^2+^ diffusion time | 0.33 ms |
| I_Ca_ density prefactor | 1.00 |
| P_Ca_ | 17.85 µmol/C/ms |
| I_Ca_ alphafca | 0.002 |
| I_Ca_ Betafca | 0.08/(1+(35/cp)^2^) |
| I_Ca_ finf | 1 – 1/(1 + exp(-(v+22.8)/9.1))/(1 +exp((v-50)/10) |
| LCS refractory period | 45 ms |
| Fluo4 dye buffer concentration | 40 µmol/L |
| RyR2 transition rate CASQ2 Unbound>Bound | 4.00 ms^-1^ |
| RyR2 transition rate CASQ2 Bound>Unbound | 0.40 ms^-1^ |
| RyR2 Ca^2+^ sensitivity | 10 µmol/L |

Table S 1 Computer model parameters modified from Hwang et al. (2020). cp, Ca^2+^ in proximal space.

**Supplementary References**

1. Choi BR, Li W, Terentyev D, Kabakov AY, Zhong M, Rees CM, Terentyeva R, Kim TY, Qu Z, Peng X, et al. Transient Outward K+ Current (Ito) Underlies the Right Ventricular Initiation of Polymorphic Ventricular Tachycardia in a Transgenic Rabbit Model of Long-QT Syndrome Type 1. *Circ Arrhythmia Electrophysiol*. 2018;11:e005414.
2. Hwang J, Kim TY, Terentyev D, Zhong M, Kabakov AY, Bronk P, Arunachalam K, Belardinelli L, Rajamani S, Kunitomo Y, et al. Late INa Blocker GS967 Supresses Polymorphic Ventricular Tachycardia in a Transgenic Rabbit Model of Long QT Type 2. *Circ Arrhythm Electrophysiol*. 2020;13:e006875.
3. Zhong M, Rees CM, Terentyev D, Choi BR, Koren G, Karma A. NCX-Mediated Subcellular Ca2+ Dynamics Underlying Early Afterdepolarizations in LQT2 Cardiomyocytes. *Biophys J*. 2018;115:1019–1032.
